# Feeding the machine: Challenges to reproducible predictive modeling in resting-state connectomics

**DOI:** 10.1162/netn_a_00212

**Published:** 2022-02-01

**Authors:** Andrew Cwiek, Sarah M. Rajtmajer, Bradley Wyble, Vasant Honavar, Emily Grossner, Frank G. Hillary

**Affiliations:** Department of Psychology, Pennsylvania State University, University Park, PA, USA; Social Life and Engineering Sciences Imaging Center, Pennsylvania State University, University Park, PA, USA; College of Information Sciences and Technology, Pennsylvania State University, University Park, PA, USA; Rock Ethics Institute, Pennsylvania State University, University Park, PA, USA; Institute for Computational and Data Sciences, Pennsylvania State University, University Park, PA, USA

**Keywords:** Machine learning, Classifiers, Predictive modeling, Brain networks, Clinical neuroscience

## Abstract

In this critical review, we examine the application of predictive models, for example, classifiers, trained using machine learning (ML) to assist in interpretation of functional neuroimaging data. Our primary goal is to summarize how ML is being applied and critically assess common practices. Our review covers 250 studies published using ML and resting-state functional MRI (fMRI) to infer various dimensions of the human functional connectome. Results for holdout (“lockbox”) performance was, on average, ∼13% less accurate than performance measured through cross-validation alone, highlighting the importance of lockbox data, which was included in only 16% of the studies. There was also a concerning lack of transparency across the key steps in training and evaluating predictive models. The summary of this literature underscores the importance of the use of a lockbox and highlights several methodological pitfalls that can be addressed by the imaging community. We argue that, ideally, studies are motivated both by the reproducibility and generalizability of findings as well as the potential clinical significance of the insights. We offer recommendations for principled integration of machine learning into the clinical neurosciences with the goal of advancing imaging biomarkers of brain disorders, understanding causative determinants for health risks, and parsing heterogeneous patient outcomes.

## BACKGROUND

In settings where large amounts of well-characterized training data are available, modern machine learning (ML) methods offer some of the most powerful approaches to discovering regularities and extracting useful knowledge from data (Bishop, 2006; Goodfellow et al., 2016; Hastie et al., 2009; Mitchell, 1997). Of particular interest are algorithms that, given a data set of labeled samples, learn a predictive model, for example, a classifier, for labeling novel samples drawn from the same distribution as the training data. Programs for training such classifiers typically optimize a desired objective function on a given set of training samples. Advances in ML have revolutionized the design of systems for natural language processing (Manning et al., 2014; Mikolov et al., 2013; Turian et al., 2010), computer vision (Bradski & Kaehler, 2008; Deng et al., 2009; Forsyth & Ponce, 2002), network analysis (Hamilton et al., 2017), and bioinformatics (Baldi et al., 2001; Larrañaga et al., 2006; Min et al., 2017). A number of publicly available ML libraries (e.g., Scikit-learn, TensorFlow) can now be deployed, permitting “off-the-shelf” application of these analyses for a number of data types including behavioral, genetic, and imaging data (Abadi et al., 2016; Abraham et al., 2014).

In one sense, predictive models trained using ML are like traditional statistical models, for example, regression: there are covariates, an outcome, and a statistical function linking the covariates to the outcome. But where ML algorithms add value is in handling enormous numbers of features or predictors, heterogeneous data types (e.g., images, text, genomic sequences, molecular structures, networks, and longitudinal behavioral observations), and combining them in complex, nonlinear ways to make accurate individualized prediction, that is, a clinical diagnosis. This review examines the use of predictive models in ML and resting-state connectomics with focus on several particularly important issues, including “overfitting” and its related consequences, sample size and implications for modeling clinical heterogeneity, and methodological transparency.

### Prediction Modeling in the Neurosciences

There has been growing use of ML to determine if brain network metrics can serve as classifiers of brain disorders with several high-profile reviews recently published (Bassett et al., 2020; Braun et al., 2018; Parkes et al., 2020; Vu et al., 2018). Many of the canonical networks identified in rsfMRI studies (e.g., default mode network) have been of critical focus in studies of large-scale network plasticity in a range of brain disorders including schizophrenia (de Filippis et al., 2019; Lefort-Besnard et al., 2018; Progar & May, 1988; Steardo et al., 2020), autism (L. Chen et al., 2020; Glerean et al., 2016; Hegarty et al., 2017), Alzheimer’s disease and related dementias (Langella et al., 2021; Pellegrini et al., 2018; Salvatore et al., 2015), and brain injury (Bonnelle et al., 2012; Caeyenberghs et al., 2017; Gilbert et al., 2018; Roy et al., 2017).

While the high dimensionality of functional imaging data—relationships between hundreds or thousands of time series observations—may push the limits of traditional modeling, ML approaches can capitalize on the complexity of multimodal datasets (Baltrušaitis et al., 2019; Gao et al., 2020; Guo et al., 2019) and provide opportunity to examine interactions among variables otherwise impossible to test. Therefore, there is evident potential for the application of ML to incorporate a wide array of data structures into prediction modeling including behavioral, brain imaging, physiological measurements, and genetic markers.

### Growing Pains in ML and Resting-State Connectomics

Perhaps the most common methodological concern in applied ML is overfitting, or training an algorithm to predict with very high accuracy features within a single dataset at the expense of predicting a phenomenon more generally (Dietterich, 1995; Ng, 1997; Roelofs et al., 2019; Srivastava et al., 2014). Overfitting has profound implications for reproducibility, portability, and generalizability of findings. Importantly, the difficulty of preventing overfitting is underappreciated, and even typical remedies, such as cross-validation, can allow for analysis hyperparameters to become tuned, or “overhyped,” to a specific set of data (Hosseini et al., 2020; Poldrack et al., 2020). These concerns underscore the need for greater transparency in model selection, enforcement of model parsimony, and rigorous testing and validation of trained models on independent validation data, with attention to class imbalance in the data, relative costs of false positives versus false negatives, and the tradeoffs between them (Varoquaux et al., 2017).

Related to overfitting are concerns about the size or heterogeneity in the training and test samples (Poldrack et al., 2020). When a sample is overly restrictive along dimensions that influence outcome in neurological disorders (e.g., severity of disease, age of onset), it may reduce the study reproducibility and the ability to predict the phenomenon as it naturally occurs (Caruana et al., 2000; Hawkins, 2004; Schaffer, 1993; Srivastava et al., 2014). As an example, an investigator may have access to a large database of cases of individuals diagnosed with a neurological or psychiatric disorder that can be used for training and test datasets. Even with conservative training and only single exposure to the testing dataset (the gold standard), the result may not generalize if the sample is restricted in its range of characteristics with respect to demography, symptom severity, or disease/injury chronicity.

### Goals of this Review

There is significant and justified enthusiasm for using ML approaches to advance our understanding of brain disorders. With the ever-increasing application of ML in the study of resting-state connectomics, the importance of the implementation of and adherence to best practices is further underscored. Given this backdrop, we review 250 papers using ML for diagnosis or symptom profiling of brain disorders using resting-state fMRI methods, coding information regarding the methods used with particular focus on how algorithmic “success” was determined, the use of a lockbox dataset (i.e., a data set that can be accessed only once at the end of the analysis, also called a holdout set, a test set, or an external set), transparency in the approach, sample size and heterogeneity, and the types of conclusions drawn. We aim to provide a summary of the state-of-the-art in ML applications to one area of clinical neuroscience with the goal of identifying best practices and opportunities for methodological improvement. While we focus on resting-state fMRI connectomics here, the issues addressed likely have relevance for a wider range of ML applications in the neurosciences.

### Method: Literature Review

We conducted a literature search using the following search terms in the PubMed database: (ML OR classifier OR supervised learn OR unsupervised learn OR SVM) AND (brain) AND (network OR graph OR connectivity) AND resting AND (imaging) AND (neurological OR clinical OR brain injury OR multiple sclerosis OR epilepsy OR stroke OR CVA OR aneurysm OR Parkinson’s OR MCI or Alzheimer’s OR dementia OR HIV OR SCI OR spinal cord OR autism OR ADHD OR intellectual disability OR Down syndrome OR Tourette) AND “humans”[MeSH Terms].

We did not bound the date range for our search, but we excluded non-English papers, review papers, and animal studies. We also excluded papers that were based on simulations or other nonhuman data. Our initial search returned 471 papers that were reviewed for inclusion. Two reviewers independently screened all of the papers returned from the above search at the title and abstract level for exclusionary criteria.

By examining each paper title and abstract, papers were excluded based on the following categories: (1) examined structural brain imaging only (*n* = 98; 21%); (2) did not examine a clinical phenomenon (*n* = 59; 13%); (3) focused on automated tissue segmentation or lesion identification (*n* = 48, 10%); (4) was focused on algorithm or method development without clinical diagnostics (*n* = 41, 9%); (5) used other imaging approaches such as EEG/MEG (*n* = 33, 7%); (6) did not implement formal network analysis (*n* = 27, 6%); (7) was not an empirical study, including reviews and perspectives (*n* = 25, 5%); (8) did not use machine learning (broadly defined) or classification (*n* = 13, 3%); or (9) another reason consistent with the exclusionary criteria (*n* = 9, 2%). This resulted in exclusion of 353 papers, and for the remaining 118 papers (25%) the full paper was included in the final analysis. For the full-text review, two reviewers were assigned to each specific section based on their respective specialties and completed a full analysis on multiple papers to identify any potential inconsistencies between the reviewers. Following this brief training for inter-rater consistency, the reviewers completed a full analysis of the papers independently.

Based on feedback during the review process, we broadened our review to include terms sensitive to papers using deep learning approaches. A second identical keyword search to the above was conducted, while inserting the following terms to capture ML and deep learning approaches:

“(deep learn* OR deep belief network OR multilayer perceptron OR autoencoder OR convolution neural network OR artificial neural network OR generative adversarial network OR machine learning OR ML OR classifier OR supervised learn OR unsupervised learn OR SVM) AND …”).

**Figure 1.**
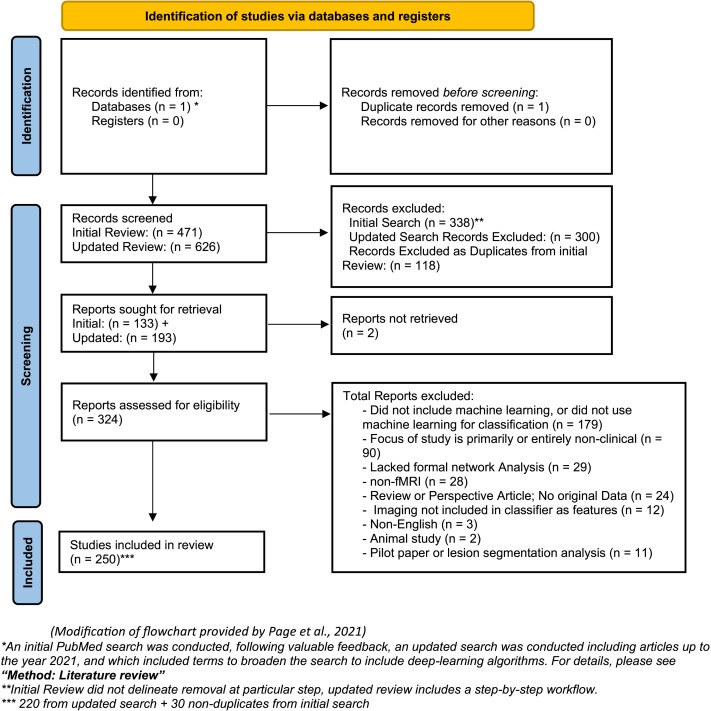
PRISMA flowchart of literature review. *An initial PubMed search was conducted, following valuable feedback, an updated search was conducted including articles up to the year 2021, and which included terms to broaden the search to include deep learning algorithms. For details, please see section Method: Literature Review. **Initial Review did not delineate removal at particular step; updated review includes a step-by-step workflow. ***220 from updated search + 30 nonduplicates from initial search. Modification of flowchart provided by Page et al. (2021).

The second search (April 2021) revealed 625 papers and based on abstract review (or full manuscript review if necessary), 405 papers were excluded based on the following categories and several for multiple reasons: (1) did not use machine learning (broadly defined) or classification (179, 28.6%); (2) did not examine a clinical phenomenon (*n* = 90, 14.5%); (3) did not implement formal network analysis (*n* = 29, 4.6%); (4) used other imaging approaches such as EEG/MEG/PET (*n* = 28, 4.4%); (5) reviewed already existing literature, no new analysis (*n* = 24, 3.8%); (6) fMRI data were not included for prediction modeling (*n* = 22, 3.5%); (6) analysis included structural neuroimaging only (*n* = 12, 1.9%); (7) prospective study or proposal (*n* = 6, .009%); (8) study not available in English (*n* = 3, .004%); (9) animal studies (*n* = 2, .003%); and (10) other reasons consistent with the exclusionary criteria (e.g., pilot studies, lesion segmentation studies, *n* = 11, .018%). This resulted in retention of 220 papers from our second search (*n* = 625). After eliminating redundancies with the outcome of the initial search (*n* = 471, *n* = 118 included), the final review included 250 unique papers for analysis. A flowchart for the literature review is provided in Figure 1.

### Data Coding

To understand the methodological factors shaping machine learning use, the type of classification algorithm utilized, subject population count, and the use of permutation testing with blind analysis, as defined by Hosseini et al. (2020), were collected. Additionally, key information pertaining to the description of features input into the algorithm, the classifier design, and the performance reporting metrics chosen to measure chosen ML technique’s findings were collected. In addition to the year of publication, specific demographic factors of the participants used in each paper were recorded. These factors include age, years of education, handedness, age of diagnosis (where applicable), and socioeconomic status. Features used to train the algorithm were recorded including the use of network metrics, behavioral data, injury or disease characteristics, genetic information, blood biomarker information, medical history, and demographic factors. For network metrics specifically, information regarding the node definition and count, edge definition, and whole-brain versus subnetwork analysis were additionally recorded.

Elements shaping the result reporting of the classifier, including the metrics chosen by the article, the type of cross-validation technique, ablation reporting, and use of a lockbox (i.e., a strictly observed separation between data used to train/optimize the analysis parameters and data used to assess generalizability; see Hosseini et al., 2020) were a primary focus of this review. Because classifier accuracy was a focus for our review, this was coded for all papers, and in the case of multiple analyses, the test with the highest performance at the most stringent validation stage (cross-validation or lockbox) was selected for analysis. In cases where papers did not report total accuracy, but did report sensitivity and specificity, we calculated an accuracy value based on sensitivity, specificity, and sample size (see Supporting Information Formula: Formula S1).

The presence of a lockbox was determined through keyword search of the full text for terms such as “holdout,” “external,” “test set,” “testing,” “withheld,” or “validation-set,” followed by a critical reading of the methodology. To qualify as a lockbox, the article had to (1) set aside a subset of data for the purpose of testing the algorithm performance *following* training, (2) make explicit that no part of the data in the lockbox was included at any point during algorithm development, and (3) not report multiple training/testing phases to arrive at the final lockbox performance. From the 250 papers, 44 (16.8%) included a test dataset, and of those, 32 included both lockbox and cross-validation performance reports.

### Interpreting Model Performance

Investigators have several tools at their disposal to better understand the impact of individual features on the final performance of the algorithm. While there are myriad ways in which specific tools can be implemented, we searched for and coded the four most common methods observed in this literature set listed here in order of commonality: (1) feature importance, (2) permutation testing, (3) ablation analysis, and (4) consensus analysis.

Feature importance, or the discriminative power of an individual feature as assigned by the trained algorithm, is an inherent element of many machine learning methodologies wherein features are ranked by their relative impact on the decision boundaries set by the algorithm. An article was coded as including feature importance if it included a report of some or all top-ranking features with some quantitative analysis of their relative contribution, such as (but not limited to) Gini index, Kendall’s tau values, or the correlation coefficient *r*.

Permutation tests use thousands of randomized shufflings to simulate the distribution of possible outcomes that a given comparison could have revealed if the independent variable was meaningless with respect to the analysis (i.e., the null hypothesis distribution). This technique can then measure the likelihood of an observed analysis outcome with an observed set of data or analysis outcome. Papers that run such analyses and report the likelihood of chance performance, generally in the form of *p* values, were coded as reporting this valuable analytical technique.

An ablation analysis examines the performance of the algorithm when portions of the algorithm are removed in order to either improve performance (i.e., during training) or to determine which portions of the algorithm or dataset contribute to the algorithm’s accuracy. This is similar to feature selection in the context of neuroscience (Guyon & Elisseeff, 2003). For a paper to demonstrate an ablation report per our coding scheme, it must show the changes to performance in training, whether as a function of feature reduction or of iteration count.

Consensus analysis is another common technique for analyzing relative importance of features by way of the ratio of times a feature is selected across the number of training/validation folds. Articles providing either a raw count or some other form of occurrence frequency for key features were coded as demonstrating a basic consensus analysis.

## RESULTS

### Representation of Clinical Disorders in Review

The final review included 250 studies largely composed of case-control designs focused on prediction modeling of diagnostic accuracy. The studies ranged from traditional neurological diagnoses (Alzheimer’s disease, brain injury) to psychiatric disease (depression, anxiety), to neurodevelopmental diseases (schizophrenia, autism spectrum). A summary of the distinct clinical disorders represented in the review is provided in Supporting Information Table S1. The largest representation of studies examined Alzheimer’s disease or related dementias (*n* = 66, 26.4%), depression/anxiety (*n* = 40, 16.0%), schizophrenia spectrum disorder (*n* = 34, 13.6%), Autism spectrum disorder (*n* = 33, 13.2%), and brain injury (*n* = 15, 6.0%).

For training, sample sizes ranged from 17 to 1,305 subjects for total samples and 8 to 653 for group-level data (case-control designs). For test datasets, the sample sizes for the total population ranged from 8 to 477 subjects and group-level data ranged from 1 to 185. See Table 1 for breakdown of training and test dataset sample sizes based on population and subgroup. These sample sizes are consistent with those observed elsewhere (Poldrack et al., 2020), and we anticipate that the large majority of studies present in this review were underpowered for reliable prediction modeling, resulting in low confidence in the portability of the reported algorithm and reproducibility of the finding in other samples.

**Table 1.** Sample sizes for population and subgroups in training and test datasets

**Sample**	**Training set (*n* = 250)**		**Test set (*n* = 44)**	
	**Total**	**Subgroup**	**Total**	**Subgroup**
**Range**	17–1305	8–653	8–477	1–185
**Mean**	126.7	50.0	96.6	38.1
**Median**	77	29	39	20
**Studies with *n* ≤ 50**	80 (32.0%)	192 (76.8%)	23 (52.3%)	35 (79.6%)
**Studies with *n* ≤ 30**	24 (9.6%)	136 (54.4%)	14 (31.8%)	28 (63.6)
**Studies with *n* ≤ 20**	3 (1.2%)	82 (32.8%)	8 (18.2%)	22 (50.0%)

### Network Characteristics

Consistent with the inclusionary criteria, 100% of the studies used at least one network metric as input during classifier identification. Table 2 provides descriptive data for the types of network studies included and the characteristics of the networks analyzed. A majority of the studies used whole-brain network information as features (73%). Similar to other examinations of the use of network neuroscience to examine clinical disorders, there was a wide range of brain parcellation values, resulting in graphs of widely varying sizes and complexities (Hallquist & Hillary, 2018).

**Table 2.** Network data: Characteristics of functional brain imaging network analysis including in prediction modeling

	**Range**	**Median**	**Mean (*SD*)**	**Mode**	
Network Nodes (parcellation) *n* = 221*	<10 to 67,955	90	483.9 (6,654.5)	90	
	**Correlation (e.g., Pearson’s *r*)**	**Partial correlation**	**Multiple**	**Causal modeling**	**Other**
Edge Definition *n* = 247*	67.9%	3.2%	6.1%	3.6%	18.3%
	**Whole brain**	**Modules/subnetworks**		**Nodes/seed-based**	**Unclear**
**Scope of study *n* = 250**	73.1%	19.0%		7.9%	3%

* *Note*: All studies included defined nodes, but in some cases the exact number of nodes was unclear with respect to ML training (*n* = 30). Similarly, all studies examined connectivity between brain regions, but for a small number of studies there was no clear edge definition (*n* = 3).

### Sample Characteristics

Sample characteristics including demographics and common clinical indicators were examined. While age of the sample was commonly reported, only 25.6% of studies included a measure of time since diagnosis, fewer still reported age of diagnosis (10.8%), and few included demographic factors such as race (5.6%). Several studies lacked a “healthy control” group. In these cases, the studies either compared the same sample at two timepoints (1) or classified against two separate clinical groups (5). See Supporting Information Table S2 for details regarding reported demography.

### Classifier Information

Critical to our goal was to assess the classifiers used in this literature, the most common input features, and how the classifiers were evaluated. Table 3 provides summary information for characteristics of classifiers used across the 250 studies. Support vector machines were the most prevalent ML algorithm selected, appearing as at least one of the utilized classifiers in 68.4% of papers. The three next most common techniques used were linear discriminant analysis (8.8%), regression classification (8.8%), random forest (8.0%), and deep learning approaches (8%). In the papers reviewed, 18.8% implemented multiple ML algorithms.

**Table 3.** Classifier types, inputs, and metrics for evaluation during classification

**Classifier**	**SVM**	**RF**	**KNN**	**LOG_R**	**LDA**	**Deep learning**	**Multiple**	**Other**
Frequency*	171 (68.4%)	20 (8.0%)	17 (6.8%)	22 (8.8%)	22 (8.8%)	20 (8.0%)	46 (18.0%)	52 (20.8%)

**Inputs into classifier**	**Brain network metrics**	**Injury/disease actor**	**Demographic**	**Behavior/cognitive data**	**Medical Hx**	**Meds**	**Genes/blood biomarkers**	**Other**
**Frequency**	100%	13.5%	10.1%	5.9%	2.5%	1.7%	0%	1.6%

**Metric for evaluation**	**Accuracy**	**Sensitivity**	**Specificity**	**AUC (AUROC)**	**Predictive power**	**Regression outputs**	**Other (e.g., F1)**	
**Frequency**	87%	70.4%	69%	40%	12%	12%	20%	

*Note*: SVM, support vector machine; RF, random forest; KNN, *k* nearest-neighbor; LOG_R, logistic regression; LDA, linear discriminant analysis. *Total >100%, including studies with more than one classification approach.

The feature types chosen to be used for a classifier, while not inherent to the quality of the ML algorithm, do speak to the nuances of the aims of the collected studies. While every study collected *some* form of injury or disease characteristic (at the very least for the purpose of identifying patient and healthy control groups), roughly 8% of studies included some form of these metrics as features to include in the classifier, and even fewer included demographic information (7%) and/or behavioral or cognitive data (4%) as features for training. Medication history, current medications, or other clinical metrics were rarely included as features (<1%). Only one study utilized blood biomarkers, and none included genetic factors in addition to network metrics, revealing an as-of-yet untouched potential for more nuanced feature-pattern discovery.

Accuracy was the primary performance metric, with roughly 86.8% of papers reporting accuracy as the baseline measure of performance. More than two-thirds of studies included sensitivity and specificity, two metrics vital for identifying bias in classification, especially in the case of sample imbalance, whereas only 40.0% of studies included a full area under the receiver operating characteristics curve (AUROC) report; 12.0% of studies included predictive power, and 12.0% of studies included some form of regression analysis in addition to the classification outputs. Finally, 20.8% of studies utilized some other form of metric performance reporting, such as F1 scores; all such measures fitting the “other” category were utilized in less than 5% of papers.

### Validation Approaches

Most studies utilized some form of cross-validation, including leave-one-out cross-validation (LOOCV) (58.8%), *k*-fold (35.6%), nested approaches (11.2%), and multiple approaches (9.2%). Of note, 12 (4.8%) of the studies did not report any cross-validation use. In these cases, the authors either provided no alternative validation method (*n* = 8) or used a lockbox but no cross-validation (*n* = 4). The key diagnostic for overfitting, use of a lockbox, was only utilized in 16.8% of studies (Table 4). Of the studies using a lockbox, 81% (34/44) made clear that iterative training never permitted access to the test (lockbox) data, and 73.8% (31/44) reported accuracy results for both the training and lockbox data.

**Table 4.** Validation measures

**Validation procedures**			
	**Yes**	**No**	**Unclear**
**Cross-validation**	94.1%	4.2%	1.7%
**Lockbox**	20.3%	79.7%	0.0%
**If lockbox, compared once (*n* = 24)**	70.8%	12.5%	16.7%

### Interpreting Model Performance

Feature importance measures were the most common metric included, with nearly half of all studies including some level of quantitative analysis (47.2%). The other three common techniques for model interpretation were observed at a rate ranging between 1-in-3 to 1-in-5 papers. Permutation testing was included in 34.0% of all studies. Ablation reports were included in 27.7%, and consensus analyses were utilized in 20.0% of all studies (see Table 5). It was rare for examiners to include some form of all four approaches described here (2.8%), but about one-third of papers integrated two to three techniques (35.2%), more than a third integrated at least one method (38.4%), and finally one-fifth of papers did not conduct an analysis of feature importance (22.8%).

**Table 5.** Common techniques for enhancing model interpretation

**Model interpretation techniques**		
	**Yes**	**No**
**Feature importance**	47.2%	52.8%
**Permutation testing**	34.0%	66.0%
**Ablation analysis**	27.7%	72.3%
**Consensus features**	20.0%	80.0%

*Note*: >100% due to multiple approaches used in some studies.

### Classifier Performance

**Figure 2.**
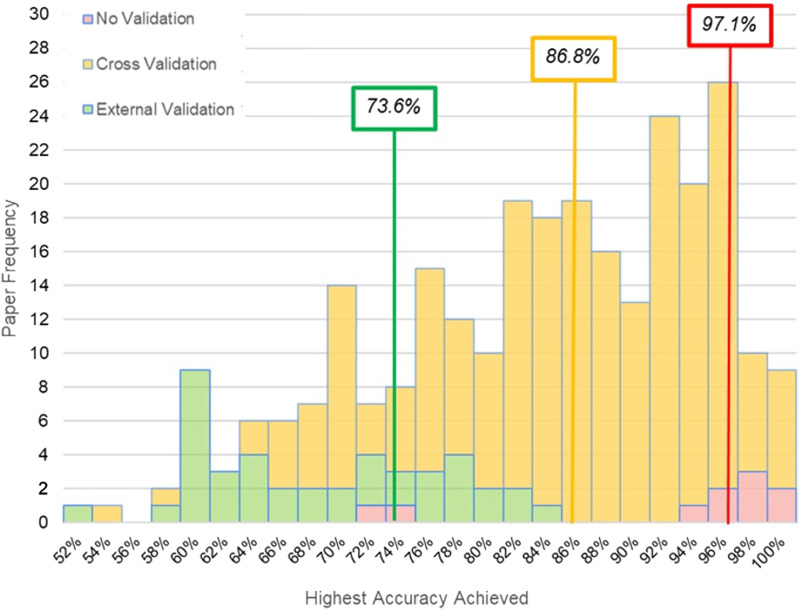
A histogram of accuracy scores for *n* = 250 studies reviewed reveals distinct distributions and median scores (organized in text boxes by color) for classification accuracy based on results using no validation, cross-validation, and external validation (i.e., lockbox).

Measuring ML performance with no form of cross-validation or lockbox validation produced a median accuracy of 97.1%. ML application using a cross-validation produced a median classification accuracy of 86.8%. When classification was performed on lockbox data, the median classification accuracy dropped to 73.9%. The distribution for accuracy values across these distinct cross-validation approaches is reported in Figure 2.

## DISCUSSION

**Figure 3.**
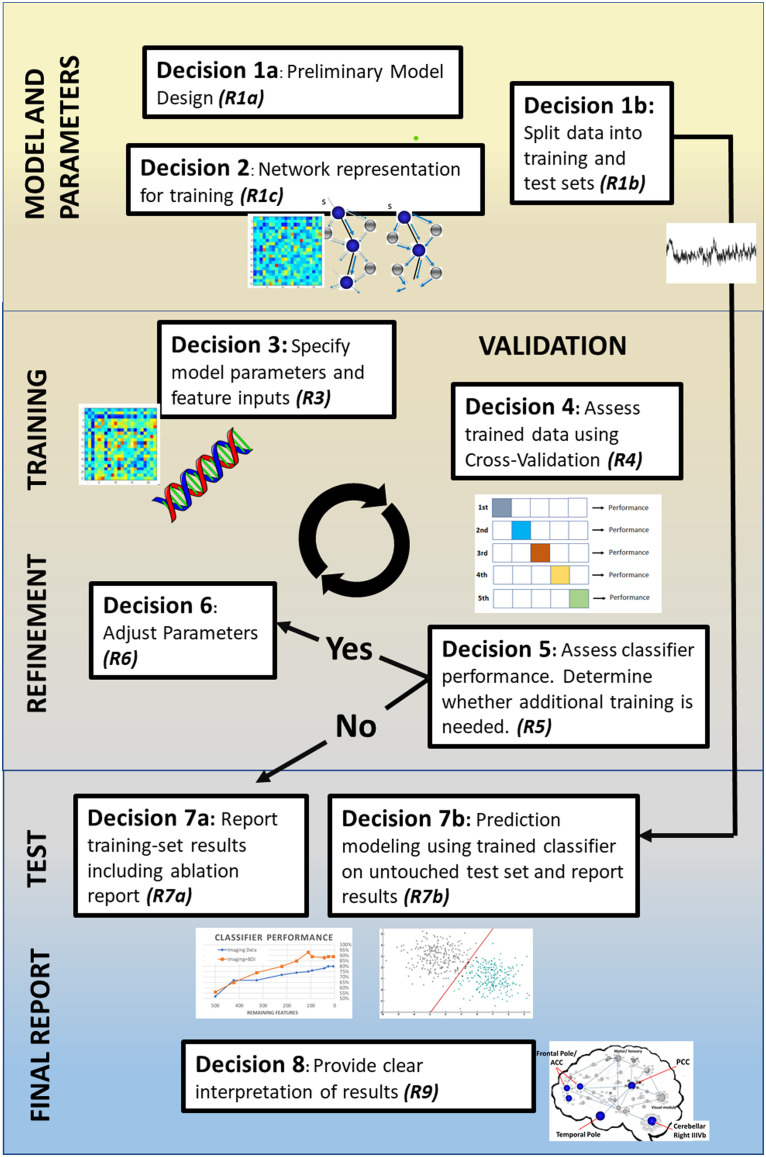
Illustration of distinct decision points in the typical ML pipeline in the papers included in this review. We identify eight distinct decision points where there are opportunities to report (**R**) information to maximize transparency. **R1a**: Justify classifier model choice from previous literature, limitations of data, and clinical goals of study. **R1b**: Explain how data were split between training and test sets (i.e., lockbox), including sample sizes and any matching of demographics or disease variables. **R2**: Make clear decisions about how the network was created, including edge definition and brain parcellation. **R3**: Make explicit the specifics of the model (e.g., parameter settings, kernel functions). Make clear which features (e.g., network metrics, clinical variables) are included in the model. **R4**: Report cross-validation method selection and implementation; justify use in context of sample size and potential risk of performance overestimation. **R5**: Explain the conditions necessary to terminate algorithm training, such as target performance or minimal feature count. **R6**: Make explicit the hyperparameter settings and any manual tuning of parameters between training iterations. **R7a**: Report training set results, including model performance, feature weights, and feature counts across training iterations. **R7b**: Explicitly state that preprocessing is unchanged from the final algorithm derived from training and that during training there was no access to the lockbox; provide the final averaged cross-validation performance and feature importance for the test set. **R8**: Provide clear interpretation and explainability for the model by highlighting any key findings in context of potential clinical utility (i.e., relevant regions of interest’s connectivity patterns).

While our review confirms the exciting promise of ML approaches in the network neurosciences to advance overall understanding of brain disorders, there also appears to be room for methodological growth. We first make several observations regarding clinical sampling and how network neuroscience has been implemented in this literature as inputs for predictive modeling. We then focus the remainder of the discussion on critical issues that, if addressed, can bring greater precision to the use of ML in the neurosciences and ideally accelerate our understanding of the pathophysiology of brain disorders. In the following we highlight several issues in order to foster discussion in the literature: (1) need for uniformity in the creation of neural networks for prediction, (2) issues of sample size and heterogeneity, (3) need for greater transparency of methods and reporting standards, (4) the focus on classification accuracy at the expense of other information, and (5) explainability and feature importance. We outline these concerns and link them to eight decision points in the typical ML processing stream outlined in Figure 3, which serves as a roadmap for key considerations and reporting opportunities at each step of the training process with the goal of improving the interpretability, reproducibility, and clinical utility.

### Sample Sizes and Clinical Heterogeneity

Roughly one-third of the studies sampled in this review had no more than 50 subjects in their total sample size for use of training and internal validation of their results. Furthermore, half of all lockbox sets examined had subgroup sample sizes of 20 or less. Thus, roughly half of the studies reviewed were likely underpowered to capture the stage, severity, and symptom constellation evident in heterogeneous neurological and neuropsychiatric disorders. Moreover, small samples likely contributed to the use of LOOCV (58.8%) instead of *k*-fold (35.6%), which may be more representative of the dataset (Poldrack et al., 2020).

Clinical characteristics of the participants (representativeness) that comprise a sample may be just as vital as the sample size. Most neurological disorders maintain heterogeneous presentations. For example, over a quarter of the studies focused on either schizophrenia or autism, both understood as existing on a “spectrum” of symptoms, which speaks to the wide range in clinical presentations (Hiremath et al., 2021; Kraguljac et al., 2021). Traumatic brain injury, as another example (6% of the studies here), varies in symptomatology, mechanism and location of injury, and severity and factors such as age at the time of injury and time postinjury. All of these independent factors may have profound consequences for neural systems and patient functioning (LaPlaca et al., 2020). To this point, few studies provided critical details regarding their samples to help address representativeness including education (35.6%), time since diagnosis (25.6%), age at diagnosis (10.8%), and race (5.6%) (see Supporting Information Table S2). The lack of clinical/demographic detail is of critical concern because even perfect prediction modeling by a classifier will leave open the question as to how the results will generalize to other samples and undermines relevance for understanding clinical pathology.

Modern data-sharing resources provide one opportunity to facilitate generalizable results by permitting clinical feature-dependent subgrouping. ENIGMA (Thompson et al., 2014, 2020), ADNI (Jack et al., 2008), ADHD200 (Di Martino et al., 2014), and OpenNeuro (Markiewicz et al., 2021) are all leading examples of data-sharing consortia that increase diversity of data collection sites, boost samples sizes, and enable representation clinical subgroups with respect to pathology chronicity and severity. While data sharing between sites poses challenges with respect to data harmonization (Radua et al., 2020), these factors (site/method) can be considered as features in prediction modeling.

### Brain Networks as Classifiers of Disease

In network neuroscience, one of the biggest challenges is determining what the network should look like, including the number of nodes and how to define the links between them. This problem is no less evident in prediction modeling, where the machine is constrained by the complexity (or simplicity) of the representative neural network used for training. There has been much recent work and emerging consensus regarding best practices for fMRI data preprocessing (Esteban et al., 2019; Nichols et al., 2017; Zuo et al., 2019) and guidance for how networks might be reliably constructed and compared (Hallquist & Hillary, 2018; van den Heuvel et al., 2017; van Wijk et al., 2010). Even so, there remains a wide range of applications of network approaches and flexibility in workflows (i.e., *investigator degrees of freedom*; Gelman & Loken, 2014), which was evident in the current sampling of the literature. Just as one example, and consistent with the review by Hallquist and Hillary (2018), there was an enormous range in brain parcellation approaches with the number of nodes ranging from <10 to over 67k (see Table 2). The number of nodes in any network is a fundamental determinant for the downstream network characteristics such as path length, local clustering, degree, and even network strength (Bullmore & Bassett, 2011; Bullmore & Sporns, 2009; van Wijk et al., 2010). Similarly, decisions about network sparsity and edge definition (e.g., correlation, partial correlation) hold crucial consequences for sensitivity to the clinical pathology (Figure 2, Decision 2). To address this issue investigators have actively modeled a range of network parameters (e.g., distinct brain parcellation approaches, distinct edge definitions) and data processing steps as part of prediction modeling to simultaneously advance the methods and isolate the role of investigator data processing decisions on model performance (Abraham et al., 2017; Badea et al., 2017; J. Chen et al., 2021; Rubbert et al., 2019).

Examiners might be best advised to test distinct network thresholds and parcellations and share study-specific workflows (Botvinik-Nezer et al., 2020) with the goal of quantifying how choices made during data processing or network definition directly influence ML parameterization. Again, these decisions require explicit reporting so that consensus can be reached regarding best practices for using networks as inputs in prediction modeling (see Figure 2, Decision 2). Finally, studies of resting-state connectomics would likely benefit from recent machine learning advances in network representation learning (F. Chen et al., 2020; Hamilton et al., 2017) and predictive modeling from longitudinal data (Hsieh et al., 2020; Le & Honavar, 2020; Liang et al., 2020a, 2020b). There is also growing application of end-to-end deep learning methods with a range of uses including data preprocessing (see Lu et al., 2021), dynamic connectivity modeling (see Fan et al., 2020), and structural connectomics (Sarwar et al., 2020).

### Transparency and Reporting Standards for Methods

It was our original intention in this review to examine indicators of overfitting, feature engineering, hyperparameter determination, and other key decision points in ML. This goal was abandoned during our review because so few papers made transparent *all* of their steps during their training and classifier identification, decisions that should be consistently reported. The absence of these details for most studies is crucial and highlighted as a primary concern expressed in work by Hosseini et al. (2020), where a clear unintentional pathway to overfitting occurs in hyperparameter development, which permits pretraining exposure to data.

Thorough reporting of the methodology surrounding the development of the ML process is key to understanding the value of the study and to allow meaningful replication analysis. For example, the reasons for algorithm selection and development, as well as the decisions made during training, can significantly impact the resultant performance and risk for overfitting (Cawley & Talbot, 2010; Hosseini et al., 2020). How features were selected should be shaped by the goals of the researcher and can shape the results of the experiment (Chandrashekar & Sahin, 2014), so the details of this step and its iterations require clear explanation in the method (Figure 2, Decisions 3 and 6). This will include, but is not necessarily limited to, the validation process, conditions for termination of the training loop, hyperparameter settings, any regularization or cost functions, and the iterative feature reduction techniques and parameters (Figure 2, Decisions 4, 5, and 6, respectively).

We propose that the reporting opportunities (**R**) presented in Figure 2 represent the standard minimum to assess how ML training was conducted and how feature inputs were selected. These questions, left unanswered, prevent an honest determination of overfitting risk and study generalizability in the absence of replication. There is real need in the literature reviewed here, and perhaps in others where prediction modeling is being used, to increase methodological transparency. To list the decisions made in the machine learning processing stream, and provide subsequent theoretical grounding for each, enables critical review of the algorithm. In addition, providing open access to the code before publication (Figure 2, Decision 3) not only enables greater replicability, but further allows for auditing of code, improving the chance of catching errors early.

### Classifier Performance: The Pursuit of Classification Accuracy

One of the most important issues to address in this literature is the emphasis on maximizing classification accuracy. It must first be recognized that purely “black box” prediction has value, even where the goal is to maximize classification with little inference as to the reasons for algorithmic success (LeCun et al., 2015), and that there is often a natural trade-off between prediction and explanation (see Yarkoni & Westfall, 2017). The perspective in the current paper, however, is that to advance our understanding of brain disorders, neuroscientific theory must set the guiderails for predictive modeling and interpretation beyond prediction holds important value.

To provide the logic for this perspective, we might imagine a scenario where clinical investigators implement a highly complex deep learning algorithm to classify “responders” for a new drug for a brain disease. While there is immediate clinical value in a completely “black box” classifier that accurately separates responders from nonresponders, the algorithm affords no additional *understanding* of the disease. In this scenario there is no new information about why responders benefited from the treatment and, importantly, why nonresponders did not—information that can be used to improve both future prediction models and clinical interventions. Ultimately, prediction modeling exists on an “explanation-prediction” continuum with some loss in transparency as one moves from less complex inferential models to more opaque models that are designed to optimize predictive performance (see Bzdok & Ioannidis, 2019). Investigators must strike a balance between ever-increasing algorithmic complexity and the need for understanding underlying mechanisms.

Given this perspective, there are three issues to consider with respect to the focus on prediction accuracy in this literature. First, there was a nearly 15% drop-off in the performance from cross-validation test (i.e., *internal*) to lockbox performance. The reduced classification accuracy from training to test data set is unsurprising, but makes clear what has been known for some time: lockbox sets should be required in any published study in order to guard against overfitting and maximize generalizability (Poldrack et al., 2020). We anticipate that overfitting is at least partially a consequence of the current overreliance on accuracy as the primary measure of an algorithm’s performance, but it also highlights an opportunity to improve machine learning practices.

A second, and related, issue is that classification accuracy achieved in the absence of lockbox data (83% of the studies reviewed) presents the field with inflated validation results that become unrealistic benchmarks for others to match. In the current review, authors commonly compared accuracy of their work with known standards maintaining that it “outperformed” other algorithms. These comparisons have been formalized elsewhere in national and international competitions where investigators have been invited to apply ML to imaging data to accurately diagnose disorders such as schizophrenia (see Silva et al., 2014) and ADHD (see Brown et al., 2012). As outlined in a number of reviews, it is likely premature to believe that machine learning can currently fill any needed void as the primary diagnostic criterion for brain disorders (Mei et al., 2020; Pellegrini et al., 2018; Zhang-James et al., 2020). It is also unreasonable to assume that any single data classification algorithm will differentiate one group from another with near-perfect reliability, with the exception of severe clinical phenomena that are readily dissociated with standard clinical measurement (e.g., schizophrenia). Where classification can make a crucial impact, however, is by uncovering interactions between features that lead to novel pathways of discovery and intervention in the clinical neurosciences (more on this below).

Finally, accuracy can be bolstered by additional metrics including sensitivity, likelihood ratio, log loss, and the AUROC are a good first step for their ability to expand on the base information given by accuracy (Poldrack et al., 2020). This added granularity uncovers potential weaknesses of a given model, such as high degrees of type 1 or 2 errors, two issues that can be obscured in imbalanced datasets.

### Adding Context to Performance in Machine Learned Models

In moving beyond classification performance, ML offers unparalleled opportunities to gain new insights into *how* neural systems adapt to brain injury and disease through use of techniques that highlight the contribution of the features to the algorithm’s performance and the significance of the model’s predictive ability. These measures add transparency to the training process (Sheikholeslami, 2019) and may help to uncover key underlying neural substrates previously unknown to the clinical field (Fawcett & Hoos, 2016). Furthermore, specific approaches, such as ablation studies, can even be used to identify new insights, essential aspects, and functional redundancies that contribute to the robustness of a given algorithm (Meyes et al., 2019). Some of the most commonly used machine learning algorithms in the field (SVM, RA) can integrate and appropriately weight the contributions of different features (Figure 3, Decision 7a, 7b). Despite this, use of these valuable tools remains limited, as highlighted in Table 4.

Feature importance was the most commonly observed technique within the reviewed literature, yet was implemented in less than half (47.2%) of studies. Similarly, measures that increase the robustness of the findings, such as determining consensus features (features that have been repeatedly selected across training iterations), were only adopted in a fifth (20.0%) of examined studies. Both of these methods enable the reporting of the relative discriminative ability of specific features. Doing so allows clinical researchers to highlight patterns of specific importance that can be linked to disease and injury phenotypes.

Ablation reports, included in roughly one-third (34.0%) of studies, examine the relative contribution of an individual feature (or set of features) on classification accuracy through selective removal and reanalysis. This metric is valuable for understanding when there are diminishing returns from overselecting features or for establishing a desired trade-off for model complexity against performance. Inclusion of an ablation report not only highlights the stability of algorithm performance, but also can establish baselines for necessary model complexity for reliable diagnosis of a given condition.

Finally, by repeated randomization of class labels through a permutation analysis (used in 27.7% of studies), the risk of the final feature set being discriminative by chance alone can be assessed through a computed significance level. Such analyses measure the reliability of the feature set used for classification.

There are tools that are available to investigators that permit stronger inferences about the reasons for classification success, though they were not commonly used in the papers reviewed here. A model in the absence of interpretation limits the ability for clinicians and researchers to build targeted treatments or identify risk factors that can be used to advance clinical. Understanding the contribution of specific features to classification success enables better stakeholder (e.g., neuropsychologists; neurologists) involvement concurrent with the development of models on the front end (Hedderich & Eickhoff, 2021) as well as explicit techniques to provide a clear explanation of the output of the classifier to the neuroscientist or physician at the back end (Heinrichs & Eickhoff, 2020).

## CONCLUSION

Computational neuroscience holds the promise of fundamentally changing our understanding of brain disorders, and, with this promise, comes much deserved enthusiasm. However, the use of ML techniques in network neuroscience reflects the growing pains observed when novel methods capture the excitement of a research community, something that the field of functional brain imaging has experienced before (Kosslyn et al., 1995; R. A. Poldrack et al., 2020; Vul et al., 2009). The ultimate goal for clinical neuroscience is to understand how the brain adapts to injury and disease, and ML can be leveraged to help uncover critical interactions between behavioral, genetic, blood-based biomarkers, imaging and other measurements of neural systems. While this literature review revealed examples where ML was being used in ways that may advance our understanding of clinical neuropathology, there is significant need for greater methodological transparency, better representation of heterogeneous clinical disorders in the training and testing datasets, and greater devotion to understanding mechanisms of neuropathology as opposed to binarized diagnostic success. In the case of the latter, there does appear to be a consequence for overemphasizing classification accuracy both in method and outcome. The generalizability of the results, replicability of the methods, and clinical value gained by the work can then be the guiding principles for our work. We have offered a few conservative recommendations in this review with the goal of continuing a dialog regarding how we can transition toward a more mature integration of ML into the neurosciences that accelerates our understanding of brain disorders and ultimately improves patient outcome.

## SUPPORTING INFORMATION

Supporting information for this article is available at https://doi.org/10.1162/netn_a_00212.

## AUTHOR CONTRIBUTIONS

Andrew Cwiek: Conceptualization; Data curation; Formal analysis; Investigation; Methodology; Writing – original draft; Writing – review & editing. Sarah Rajtmajer: Conceptualization; Investigation; Methodology; Resources; Writing – original draft; Writing – review & editing. Bradley Wyble: Conceptualization; Resources; Writing – original draft; Writing – review & editing. Vasant Honavar: Resources; Writing – original draft; Writing – review & editing. Emily Grossner: Formal analysis; Investigation; Writing – review & editing. Frank Hillary: Conceptualization; Data curation; Formal analysis; Investigation; Methodology; Project administration; Visualization; Writing – original draft; Writing – review & editing.

## TECHNICAL TERMS

Classifier: An algorithm designed to classify two or more groups using a given set of variables.
Training: A generally iterative process wherein an algorithm is refined to better classify a subject into their correct group by tuning the parameters for selection of important variables.
Features: The variables input into the algorithm for use in classification.
Overfitting: When the algorithm is too closely attuned to the data it was trained on, to the detriment of the algorithm’s generalizability to new samples.
Dimensionality: The number of features given to the algorithm.
Cross-validation: A process to limit overfitting through repeated splitting of the data into training and testing sets to prevent overfitting.
Lockbox: A set of data intentionally set aside before training the algorithm and used exactly once after training to test the generalizability of the result.
Permutation testing: A method for testing the final feature set against chance performance through repeated randomization of class labels (i.e., patient vs. healthy control) and comparison of the distributed accuracy to the observed performance.
Ablation analysis: A measure of the contribution of particular variables through manual removal or addition of specific features during training.
Feature weight: The discriminative ability of a given feature as measured and quantified through various methodologies.
Consensus features: Features that are included a certain threshold of training iterations, the more training iterations that the algorithm selects the feature for, the more likely that it is discriminative for classification.
