# Supplementary material for: Feeding the machine: Challenges to reproducible predictive modeling in resting-state connectomics
Source: Netw Neurosci. 2022 Feb 1;6(1):29–48. doi: 10.1162/netn_a_00212 (PMC8942606; doi:10.1162/netn_a_00212)
Supplement: Supplementary file 1 [file netn-06-29-s001.pdf]

Cwiek, A., Rajtmajer, S. M., Wyble, B., Honavar, V., Grossner, E. & Hillary, F. G. (2022). Supporting information for "Feeding the machine: Challenges to reproducible predictive modeling in resting-state connectomics." *Network Neuroscience*, 6(1), 29–48. https://doi.org/10.1162/netn_a_00212

Supplemental Materials

Table S1: Frequency of the neurological disorders/syndromes included in this review

	Frequency	Percent
Alzheimer's disease (MCI, FTD)	66	26.4%
Mood and psychiatric (depression/ptsd)	40	16.0%
Schizophrenia	34	13.6%
Autism spectrum	33	13.2%
Brain Injury/stroke	15	6.0%
Motor disease (PD, HD)	15	6.0%
ADHD	14	5.6%
Epilepsy	13	5.2%
Health/Medical	10	4.0%
Multiple sclerosis	7	2.8%
Substance Abuse	4	1.6%
Infants/Children	4	1.6%
Other	9	3.6%
Multiple	15	6.0%

Table S2: Demographic information collected from the systematic review

	Percentage of Studies:	Count:
**Participants:**	100%	250
**Sex:**	86.8%	217
**Age:**	88.8%	222
**Left/Right Handed**	**27.2%**	**68**
**Education:**	**35.6%**	**89**
**Times Since Diagnosis:**	**25.6%**	**64**
**Age of Diagnosis:**	**10.8%**	**27**
**Race / Ethnicity:**	**5.6%**	**14**
**Socioeconomic Status:**	**0.8%**	**2**

Formula S1: Accuracy derived from sensitivity and specificity scores based upon sample size

$$Accuracy = \frac{(Sn * CG) + (Sp * HC)}{(CG + HC)}$$

Where Sn = sensitivity, CG = condition group sample size, Sp = Specificity, and HC = healthy control sample size.
